# Supplementary material for: The Systems Biology Research Tool: evolvable open-source software
Source: BMC Syst Biol. 2008 Jun 29;2:55. doi: 10.1186/1752-0509-2-55 (PMC2446383; doi:10.1186/1752-0509-2-55)
Supplement: Additional file 1 — SBRT Archive. An archive of the current version of the Systems Biology Research Tool. [file 1752-0509-2-55-S1.zip › sbrt-1.4.0/doc/developers_guide/api/sbrt/kernel/math/class-use/QuadraticExpr.html]

Uses of Interface sbrt.kernel.math.QuadraticExpr


|  |  |  |
| --- | --- | --- |
| **Overview** \| **Package** \| **Class** \| **Use** \| **Tree** \| **Deprecated** \| **Index** \| **Help** | |  |
| PREV   NEXT | **FRAMES**    **NO FRAMES**     **All Classes** |  |


---


## **Uses of Interface sbrt.kernel.math.QuadraticExpr**

| Packages that use QuadraticExpr | |
| --- | --- |
| **sbrt.ext.cplex** | Provides a class that allows communication with CPLEX. |
| **sbrt.kernel.math.optimization** | Provides classes and interfaces for creating objects relevant to solving optimization problems. |

Uses of QuadraticExpr in sbrt.ext.cplex

| Methods in sbrt.ext.cplex with parameters of type QuadraticExpr | |
| --- | --- |
| `void` | `CplexSolver.setConstraint(QuadraticExpr<? extends C> expr, Interval c)`             Sets a constraint on the provided mathematical expression of column variables. |
| `void` | `CplexSolver.setObjectiveFunction(QuadraticExpr<? extends C> objective)`             Not yet implemented. |
| `void` | `CplexSolver.setObjectiveFunction(QuadraticExpr<? extends C> objective, OptimizationSense sense)`             Sets the objective function and optimization sense to the provided values. |

Uses of QuadraticExpr in sbrt.kernel.math.optimization

| Methods in sbrt.kernel.math.optimization with parameters of type QuadraticExpr | |
| --- | --- |
| `void` | `QuadraticProgramSolver.setConstraint(QuadraticExpr<? extends C> expression, Interval c)`             Sets a constraint on the provided quadratic expression of column variables. |
| `void` | `QuadraticProgramSolver.setObjectiveFunction(QuadraticExpr<? extends C> expression)`             Sets the objective function to the provided quadratic expression of column variables. |
| `void` | `QuadraticProgramSolver.setObjectiveFunction(QuadraticExpr<? extends C> expression, OptimizationSense sense)`             Sets the objective function and optimization sense to the provided values. |

---


|  |  |  |
| --- | --- | --- |
| **Overview** \| **Package** \| **Class** \| **Use** \| **Tree** \| **Deprecated** \| **Index** \| **Help** | |  |
| PREV   NEXT | **FRAMES**    **NO FRAMES**     **All Classes** |  |


---
